# Synthesis, Optical, and Morphological Studies of ZnO Powders and Thin Films Fabricated by Wet Chemical Methods

**DOI:** 10.3390/ma13112559

**Published:** 2020-06-04

**Authors:** Robert Szczesny, Aleksandra Scigala, Beata Derkowska-Zielinska, Lukasz Skowronski, Christophe Cassagne, Georges Boudebs, Roman Viter, Edward Szłyk

**Affiliations:** 1 Faculty of Chemistry, Nicolaus Copernicus University in Toruń, Gagarina 7, 87-100 Toruń, Poland; a.scigala@doktorant.umk.pl (A.S.); eszlyk@chem.umk.pl (E.S.); 2 Institute of Physics, Faculty of Physics, Astronomy and Informatics, Nicolaus Copernicus University in Toruń, Grudziadzka 5, 87-100 Torun, Poland; beata@fizyka.umk.pl; 3 Institute of Mathematics and Physics, UTP University of Science and Technology, Al. Prof. S. Kaliskiego 7, 85-796 Bydgoszcz, Poland; lukasz.skowronski@utp.edu.pl; 4 Laboratoire de Photonique d’Angers, LPHIA, EA 4464, SFR MATRIX, UNIV Angers, 2 Boulevard Lavoisier, 49045 Angers, France; christophe.cassagne@univ-angers.fr (C.C.); georges.boudebs@univ-angers.fr (G.B.); 5 Institute of Atomic Physics and Spectroscopy, University of Latvia, 19 Raina Blvd., LV 1586 Riga, Latvia; roman.viter@lu.lv

**Keywords:** ZnO, nanoparticles, thin films, wet chemical methods, poly(vinylpyrrolidone), optical properties

## Abstract

Zinc oxide nanoparticles were prepared from Zn_5_(CO_3_)_2_(OH)_6_ precursor, capped with poly(vinylpyrrolidone) (PVP), and annealed at 600 °C. The obtained powders were characterized by a powder X-ray diffraction (PXD), transmission electron microscopy (TEM), scanning electron microscopy (SEM), UV–visible spectroscopy (UV–vis), Raman spectroscopy, infrared spectroscopy (IR), thermal analysis (TGA/DTA), and third-order nonlinear (NL) optical measurement. Morphological evaluation by TEM and SEM measurements indicated that the precursor micro-particles are ball-shaped structures composed of plates with a thickness of approximately 10 nm. ZnO thin films, as well as ZnO/polymer multilayer layouts, were obtained by wet chemical methods (spin- and dip-coating). Surface topography and morphology of the obtained films were studied by SEM and AFM microscopy. Films with uniformly distributed ZnO plates, due to the erosion of primary micro-particles were formed. The fabricated specimens were also analyzed using a spectroscopic ellipsometry in order to calculate dielectric function and film thickness.

## 1. Introduction

Through the last two decades, zinc oxide has received a broad attention due to its application in various advanced fields of science and industry such as: electronics, optics, photonics, and biotechnology. Applicability of ZnO is related to the size reduction of metal oxide structures which in turn reveals an advantageous impact on its versatile properties. ZnO is a wide band gap (3.37 eV) semiconductor with high exciton binding energy (60 meV), high thermal and mechanical stability at room temperature and piezoelectric properties [1,2]. Due to that properties ZnO is used in fabrication of nanoscale optoelectronic devices, sensors, lasers or transducers. Significantly, zinc oxide is currently extensively studied in photocatalysis as an efficient and promising material for water treatment technology [3], and in photovoltaics, as an effective material for the development of different types of solar cells [4,5,6,7]. Furthermore, ZnO nanostructures exhibit antiseptic properties, they are nontoxic, environmentally friendly, biocompatible and have been widely used in daily applications, such as pharmacy and cosmetics [8,9]. Hence, they are promising materials for production of biochemical sensors, advanced biomedical applications, or even for agricultural industry in plant protection [10,11], without risk to human health.

There are a large number of zinc oxide nanoparticle fabrication methods, such as: commercially used mechanochemical processing, physical vapor synthesis, or other laboratory scale methods (precipitation, thermal decomposition, or hydrothermal synthesis) [12]. Considering the oxide layers, they can be fabricated by physical methods such as: evaporation (molecular beam epitaxy (MBE), electron beam evaporation) or sputtering processes (e.g., magnetron and radio-frequency sputtering), and pulsed laser deposition (PLD), or by chemical techniques such as dip- and spin-coating methods, chemical bath deposition, chemical vapor deposition (CVD), plasma enhanced CVD (PECVD), atomic layer deposition (ALD), ultrasonic spray method, etc. [13,14,15,16]. Dip-coating and spin-coating deposition techniques offer the opportunity for facile fabrication of thin films of uniformly dispersed nanoparticles on the substrates, even for large areas, and allow to produce a wide variety of composite materials at mild conditions. Zinc oxide film in combination with another layer, e.g., polymer film can find many applications, including mentioned above solar cell systems. 

In this study, ZnO powders were synthesized by precipitation and decomposition of a basic zinc carbonate. The precursor powder was also utilized in the deposition process carried out using wet chemical deposition techniques. Obtained material were used to fabricate ZnO/polymer multilayer systems. For this purpose, poly(vinylpyrrolidone) (PVP) was chosen. PVP has been widely used as a biomaterial for many years in science research as well as in industry and it is known as a bulky, amphiphilic, non-toxic, and biocompatible polymer [17,18]. Furthermore, PVP is often used in nanoparticle synthesis as a surface stabilizer, growth modifier, dispersant, and reducing agent depending on the synthesis conditions [19]. The mentioned type of ZnO/PVP composites/interlayers have been fabricated and considered as the potential biosensors for superoxide anion radicals (SOR), for characterizing the antioxidant properties of fluids [20,21] and to enhance the efficiency of polymer solar cells [22]. The reports on ZnO/PVP composites have mainly described synthesis of ZnO particles capped with PVP (used as a surfactant) or preparation of composite films by solution casting technique. In both cases, the ZnO particles were homogeneously distributed in the PVP polymer matrix. Our aim was to develop a new simple and inexpensive procedure for thin film fabrication, by wet techniques, applying subsequent deposition of individual components with well separated ZnO grains. The new method provides possibility of composition control and modification by additional components, resulting in property tuning of new hybrid inorganic−organic materials.

In this study, small ZnO particles as well as ZnO/PVP layers were prepared and characterized. We have expanded the relationships between the morphology of these materials and different linear and NL optical properties as luminescence, optical limiting and optical switching responses.

## 2. Materials and Methods

### 2.1. Materials

Zn(NO_3_)_2_∙6H_2_O, NaHCO_3_ (POCh, Gliwice, Poland), and poly(vinylpyrrolidone) (PVP) K30, Mw ~ 40 000 g/mol) (SigmaAldrich, Saint Louis, MO, USA) and solvents were of analytical grade and used as purchased.

### 2.2. Synthesis of ZnO Powders

In the first stage basic zinc carbonate precursor was synthesized in reaction of Zn(NO_3_)_2_∙6H_2_O with NaHCO_3_ by slow addition of 50 mL 0.12 M NaHCO_3_ aqueous solution to the 160 mL mixture of 0.50 g Zn(NO_3_)_2_∙6H_2_O, 0.12 g PVP and deionized water, over 30 min at 70 °C, subsequently followed by 1.5 h heating, with constant stirring. Final mixture was cooled to the room temperature, the white suspension centrifuged and washed with water, ethanol, and acetone. The basic zinc carbonate was placed in a horizontal tube or muffle furnace, at the ambient atmosphere, heated at 20 °C/min or 10 °C/min rates up to 600 °C and annealed for 1 h or 2 h. The furnace was allowed to cool naturally. 

### 2.3. Thin Layer Fabrication

All films were deposited onto silicon plates (10 mm × 10 mm × 1 mm) by a spin- (Laurell 650SZ, North Wales, PA, USA) or dip-coating (Qualtech QPI-168, Denver, CO, USA) techniques. Prior to the deposition process, the substrates were thoroughly cleaned to prevent the appearance of various types of micro-contaminants in the film. The silicon wafer was pre-cleaned with organic solvents (acetone and ethanol) and three times-distilled water. On the as-prepared substrates, sonicated ethanol suspensions of precursor powders were deposited and obtained films were heated at 500–600 °C for 1–3 h. Fabricated ZnO thin films were used to prepare ZnO/PVP multilayer systems. For this purpose, PVP ethanol solution was deposited by spin-coating on ZnO/Si base using different process parameters. 

### 2.4. Characterization Methods

Powder X-ray diffractograms (PXD) were collected using using X’Pert Pro θ–2θ diffractometer (Malvern Panalytical Ltd, Malvern, UK) with CuKα radiation. Phase identification was performed by search–match procedures with an access to the ICDD powder diffraction file (PDF) and in accordance with the JCPDS cards. Scanning electron microscopy (SEM) studies were performed with a Quanta 3D FEG (FEI, Hillsboro, OR, USA) (EHT = 30 kV) instrument. Obtained powders and films deposited at Si were placed onto carbon tabs attached to aluminum SEM stubs and analyzed in the microscope without coating treatment. Transmission electron microscopy (TEM) analysis was performed on a carbon-coated copper grid and examined with a Tecnai F20 X-Twin (FEI, Hillsboro, OR, USA) instrument. Atomic Force Microscopy (AFM) analysis of films was performed using a Veeco microscope (Veeco, Plainview, NY, USA) (scan size 2–10 μm; scan rate 1 Hz, tapping mode). Thermal properties were studied by thermogravimetric-differential thermal analysis (TG-DTA) techniques, under a flowing air atmosphere using STA 409PC TG-DTA instrument (Netzsch, Selb, Germany), in the 20–700 °C range and heating rate of 5 °C/min. The FT-IR spectra of powders were collected using a FT-IR Vertex 70V (Bruker Optik, Ettlingen, Germany) spectrometer in the ATR mode in the spectral range of 100–4000 cm^−1^. Raman spectra of ZnO material were recorded using a SENTERRA II Compact Raman Microscope (Bruker Optik, Ettlingen, Germany) in the range 50–4400 cm^−1^. The Raman scattering was excited by a laser operating at 532 nm, and detected using a CCD detector. UV–vis spectra of ZnO samples were registered in solid state by diffuse reflectance spectroscopy (DRS) technique (V-750 UV–visible Spectrophotometer, JASCO, Tokyo, Japan). The band gap of ZnO was determined using modified Kubelka–Munk function. The photoluminescence properties of powders were characterized on a self-constructed equipment with Nd:YAG, LCS-DTL-374QT laser (355 nm, Russia). The spectroscopic ellipsometry (SE) technique was used to determine the optical constants and the thicknesses of prepared films. Ellipsometric azimuths, Ψ and Δ, as well as the depolarization factor (%Depol) were measured for three angles of incidence (65°, 70°, and 75°) in the NIR-Vis spectral range (413–1240 nm; 3–1 eV) by the V-VASE device (J. A. Woollam Co., Inc., Lincoln, NE, USA). 

**Figure 1:**
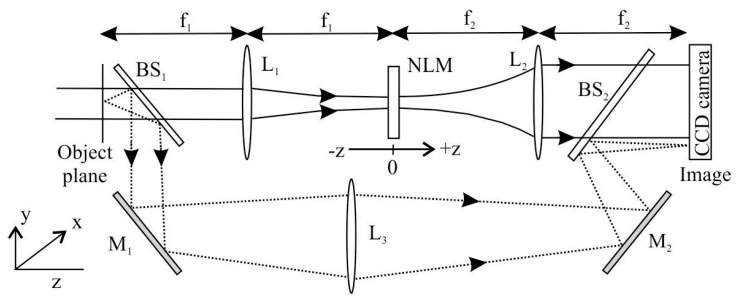
4f imaging system: L—lenses (f1 = f2); BS—beam splitters; M—mirrors.

Nonlinear optical coefficients (*n*_2_ and *β*) were determined using the D4σ Z-scan technique inside the 4f imaging system (Figure 1) by a mode-locked Nd:YAG laser (λ = 355 and 532 nm, τ = 10 ps, 10 Hz repetition rate) [23,24].

It should be noted that the beam splitter BS1, at the entry of the setup allows to monitor the fluctuations (through the lens L3) occurring in the incident laser beam, independently from the absorption that may occur inside the nonlinear material (NLM). Gaussian beam images truncated through a circular aperture (radius of 1.13 mm) at the input of the system (object plane) are registered by a single-shot CCD camera (situated in the image plane of the 4f setup) as a function of the sample position (z), while NLM moves in the focal plane. Open- and closed-aperture Z-scan normalized transmittance can be numerically processed from the obtained images, which allow simultaneous measurements of the NL refractive index (*n*_2_) and the NL absorption coefficient (*β*). In the D4σ technique, in contrast to traditional closed-aperture Z-scan method [25], the *n*_2_ value is obtained from the acquired CCD images by measuring the laser beam waist relative variations (BWRV) in the image plane. Whereas, *β* is determined using the well-known open-aperture Z-scan procedure [25,26].

The determinations of *n*_2_ and *β* are supported with simulations of the image formation inside the 4*f* system [24,27]. The classic measuring procedure considers the linear absorption coefficient (*α*) and was built to take into account the response of the material described by an effective cubic nonlinearity depending only on *β* and *n*_2_. It should be noted that thermo-optical effects are considered insignificant in the picosecond range (10 ps) and low repetition rate (10 Hz) [28]. The transmittance of the sample is

(1) $$T\left(z,u,v\right)={\left[1+q\left(z,u,v\right)\right]}^{-1/2}\exp\left[j\Delta \varphi_{\mathrm{NL}}^{\mathrm{eff}}\left(z,u,v\right)\right],$$

where q(z, u, v)=βLI(z, u, v), and $\Delta \varphi_{\mathrm{NL}}^{\mathrm{eff}}\left(z,u,v\right)={2\pi n}_{2}{\mathrm{LI}}_{\mathrm{eff}}\left(z,u,v\right)/\lambda$ — the effective value of the NL phase shift, $I_{\mathrm{eff}}\left(z,u,v\right)=I\left(z,u,v\right)\log\left[1+q\left(z,u,v\right)\right]/q\left(z,u,v\right)$ — the effective intensity, $L_{eff}=\left(1-e^{-\alpha L}\right)/\alpha$ — the effective thickness of the NLM. 

The NL phase shift can be deduced from the signals of the BWRV obtained in low and high intensity regimes. More experimental details and related data processing are described elsewhere [23,24]. ZnO suspensions were placed inside 1 mm quartz cell. It should be mentioned that the thickness of cell is much smaller than the Rayleigh range (z**_0_ = 9.5 mm) of the laser beam in the focal region. Moreover, the response of the fused silica composing the cell is considered during the measurement process in a way that the NL phase induced in the wall’s glass is deduced from the total phase measured with the solution. 

## 3. Results and Discussion

### 3.1. Synthesis and Characterization of Powders

A series of synthesis were carried out by varying the reagents molar ratio (Zn(NO_3_)_2_∙6H_2_O/NaHCO_3_: 1:1.5, 1:2., 1:3.5), the amount of PVP (0.1–1 g), heating time (0.5–1.5 h) and calcination time (1–2 h). During the synthesis process the following chemical reaction occurs [29,30]

5 Zn(NO_3_)_2_∙6H_2_O + 10 NaHCO_3_ → Zn_5_(CO_3_)_2_(OH)_6_ + 8 CO_2_ + 32 H_2_O + 10 NaNO_3_

Zn_5_(CO_3_)_2_(OH)_6_ → 5 ZnO + 2 CO_2_ + 3 H_2_O

**Figure 2:**
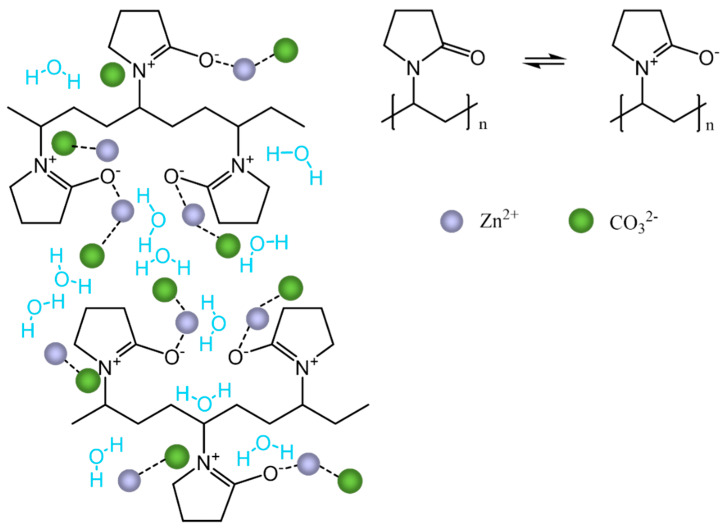
Proposed scheme of the interactions between PVP and the precursor ions.

Properties of powders obtained in typical syntheses, described in detail above, resulted in the most symmetrical and smallest particles. The particles sizes differences slightly depend on the applied reagents molar ratio and amount of capping agent (larger amounts of PVP caused aggregation of particles in bulk). PVP acts as a capping agent for metallic salts due to the steric and electrostatic stabilization of the amide and methylene groups. Proposed scheme of interactions between PVP and precursor ions is presented in Figure 2.

Most probably metal ions are bound by the ion – dipole interactions with the amide groups in the polymer chain. This effect of stabilization can be related to the precursor synthesis reaction and to the calcination process, during which PVP affects the formation of the ZnO NPs nuclei. Without the capping agent small nanoparticles with high surface energy would become larger via the Ostwald ripening process. After calcination the PVP and all undesired constituents are decomposed and pure ZnO powder is formed [31,32,33]. 

**Figure 3:**
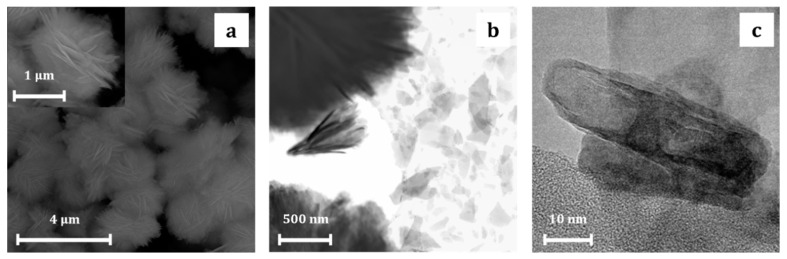
SEM (**a**) and TEM (**b**,**c**) micrographs of Zn-Carb particles obtained in typical synthesis.

Morphology of the obtained precursor micro-sized structures, in the form of balls resembling stars, was similar for all syntheses variants (Figure 3a). TEM micrographs indicate the erosion of ball-like microparticles, assisted with ultrasonification, resulted in the separated carbonate (Zn-Carb) plates with the thickness of ca. 10 nm (Figure 3b,c). 

**Figure 4:**
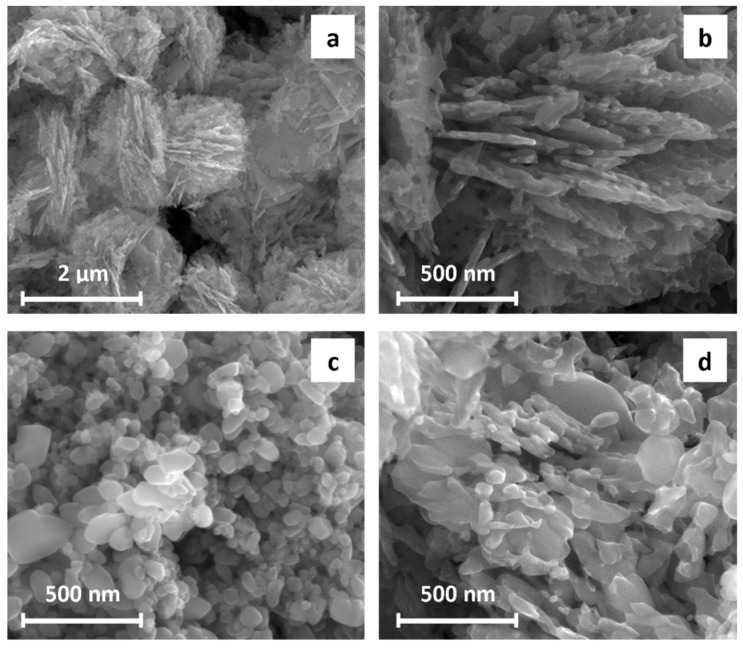
SEM micrographs of ZnO powders A type (**a**,**b**), B type (**c**) and intermediate forms (**d**).

Calcination process resulted in two main different types of powders obtained in the separate experiments at the same conditions. We have distinguished ZnO particles with well-preserved morphology of the precursor—type A (Figure 4a,b) and ZnO with modified morphology to cylindrically shaped particles of 20–50 nm width and 50–200 nm length—type B (Figure 4c). It should be noted that intermediate forms were detected as well (Figure 4d). Powder B was fabricated as a primary product in a reproducible way. Majority of the analyzes have been performed for the type B product, however a comparison of both types of powders was also examined.

**Figure 5:**
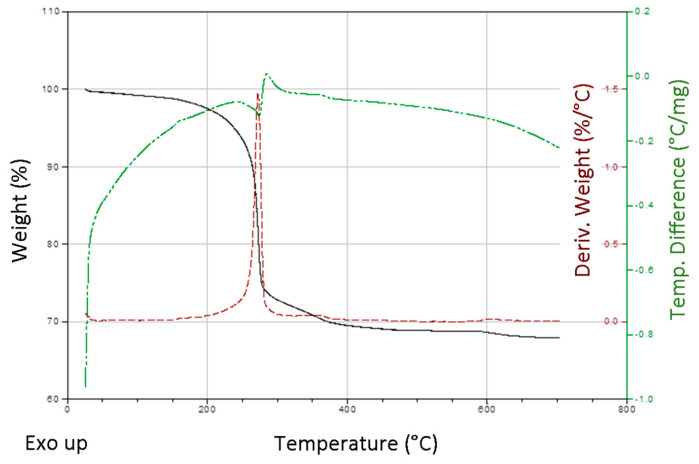
TGA/DTA thermograms of Zn-Carb precursor.

Thermal analysis measurements were carried out for the precursor obtained in non-typical synthesis, but with higher amount of PVP mass (0.5 g). The TGA/DTA curves of the precursor (Figure 5) indicates ca. 32% total weight loss in the calcination process. The first endotherm is observed in the range 25–295 °C (26.7% weight loss), while the second one between 295–490 °C (4.1% weight loss), and the third in the range 590–700 °C (0.9% weight loss). It would be difficult unambiguously identify the decomposition products in each stage due to the similar decomposition temperatures of the precursor and PVP. The amount of polymer present on the surface of precursor can be only estimated. Calculated weight loss during basic zinc carbonate decomposition process is 25.9% and corresponds to the release of carbon dioxide and water molecules. X. Wang et al. proved that thermal decomposition process of Zn_5_(CO_3_)_2_(OH)_6_ was the three-stages process with the total weight loss 26.11% [34]. According to others, thermal analysis of PVP indicated two-stages decomposition of the polymer—first observed in the range of 20–250 °C, whereas the second between 300–500 °C [17,35]. Therefore, in further studies we used a slightly higher calcination temperature (600 °C). 

**Figure 6:**
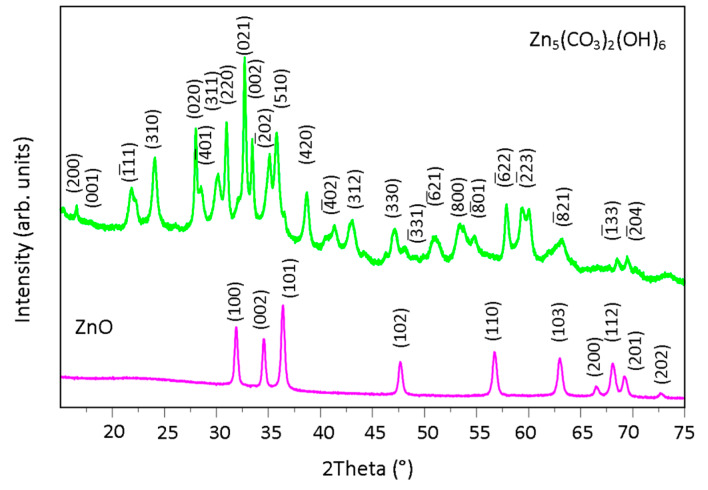
X-ray diffraction patterns of Zn-Carb (green) and ZnO (violet) powders.

Values of the precursor powders X-ray diffraction reflections recorded as 2θ angles were detected at: 12.99°, 21.85°, 24.08°, 28.02°, 30.11°, 30.98°, 32.72°, 33.46°, 35.10°, 35.79°, 38.69°, 41.34°, 43.02°, 47.13°, 51.09°, 53.37°, 54.78°, 57.87°, 59.31°, 63.19°, 68.52°, and 69.47°, pointing on basic zinc carbonate and, specifically, on hydrozincite - Zn_5_(CO_3_)_2_(OH)_6_ in accordance with the JCPDS card (no. 19-1458) [36,37,38]. The crystal faces attributed to the peaks are presented on Figure 6. Powders after calcination revealed characteristic peaks at 2θ values: 31.91°, 34.56°, 36.39°, 47.69°, 56.74°, 63.01°, 66.52°, 68.10°, 69.24°, and 72.69°, what clearly corresponds to ZnO wurtzite structure (JCPDS card no. 36-1451) (Figure 6) [37,39]. The XRD results for powders type A and B were analogous.

**Figure 7:**
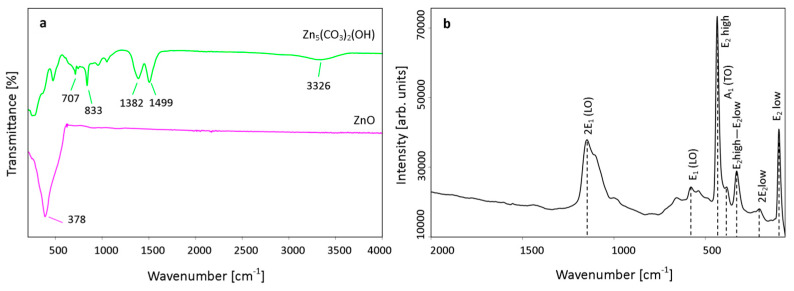
FT-IR (ATR) spectra of Zn-Carb and ZnO powders (type B) (**a**), and Raman spectrum of ZnO powder (type B) (**b**).

FT-IR spectrum of the precursor sample (Figure 7a) exhibits carbonate bands corresponding to the stretching (1382 cm^−1^, 1499 cm^−1^) and bending (707 cm^−1^, 833 cm^−1^) CO_3_^2−^ vibrations, and a broad weak band centered at 3326 cm^−1^ which can be assigned to OH stretching modes [39,40]. According to the group theory, ZnO wurtzite structure is classified to the P6_3_mc ($C_{6v}^{4}$) space group, what indicates the existence of the following optic modes: Γ_osc_ = A_1_ + 2B_1_ + E_1_ + 2E_2_. The B_1_ modes are silent, the A_1_ and E_1_ vibrations (split into transverse (TO) and longitudinal optical (LO) phonons) are both Raman- and infrared-active, whereas the E_2_ vibrations are only Raman-active [41,42]. Hayashi et al. reported the ZnO FT-IR spectrum where three distinct absorption bands between TO and LO phonon frequencies were noted [43]. However, the spectrum can be varied due to the particles morphology and three- or two-band superposition or even a broad single band was observed [44]. Here, after precursor calcination carbonate bands disappeared and characteristic broad band of Zn–O bond vibrations for zinc oxide wurtzite structure occurs at 378 cm^−1^. The Raman spectrum (Figure 7b) exhibits frequencies of the first- and the second-order Raman spectra, with the band located at 439 cm^−1^ considered as a characteristic stretching Zn-O bond vibrations of wurtzite ZnO [41]. 

**Figure 8:**
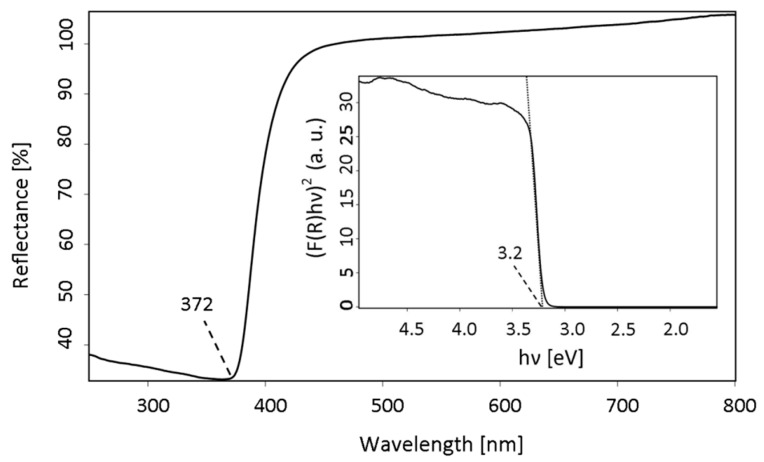
UV–vis DRS spectrum of ZnO type B powder with the inserted plot of K-M function vs. photon energy.

The UV–vis diffuse reflectance spectrum indicates sharp absorption edge distinctive for ZnO nanoparticles, observed at 372 nm (Figure 8). As the reflectance, not absorbance spectrum had been received, the band gap determination could not be performed directly from the Tauc equation and the Kubelka–Munk function had to be used for this purpose. The K-M function F(R) is directly proportional to the absorption coefficient (α) and inversely proportional to the scattering factor (S), with R is the reflectance [45]

(2) $$F\left(R\right)=\frac{\alpha }{S}=\frac{{\left(1-R\right)}^{2}}{2R}$$

For determination the band gap using Tauc relation, the following type of equation is advisable [46]

(3) $${\left(\alpha h\nu \right)}^{1/n}\approx B\left(h\nu -E_{g}\right)$$

where k is an absorption constant, Eg is the band gap and exponent n is determined by the transition type (n = 1/2 for ZnO featuring direct allowed transitions). Since the K-M function is proportional to the absorption coefficient, the relation can take the following form

(4) $${\left(F\left(R\right)h\nu \right)}^{2}=B\left(h\nu -E_{g}\right)$$

The original reflectance spectrum had been transformed by plotting the ((F(R)hv)^2^) function versus the photon energy (hv) [47]. The band gap was determined by extrapolating the slope to 0 and was amounted to 3.20 eV, similar to other reports [48].

**Figure 9:**
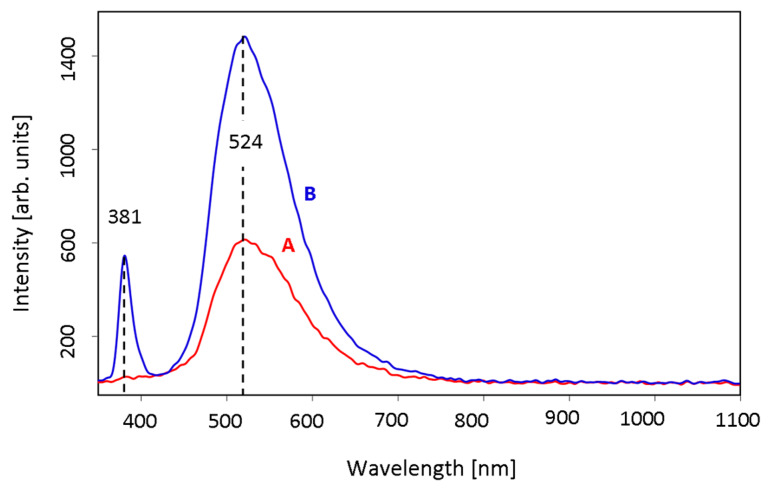
PL spectra of ZnO type A (red) and type B (blue) powders.

While the spectroscopic analysis results presented in this work were similar to different types of powders, photoluminescence measurements varies for types A and B particles (Figure 9). Intensive emission band in the UV region, observed for type B samples placed at 381 nm (shifted by 9 nm to the absorption onset), corresponds to the near band-edge emission [49]. This band noted for the A type particles, revealed quite weak intensity. Observed significant difference in bands intensities can be related to the differences in particles size. Similar phenomenon of the luminescence quantum efficiency decrease was detected, when the particles size have increased [50,51]. In this case, other factors can also cause the observed differences [52,53]. In the visible region the broad emission band at 524 nm occurs for both types of powders. Despite the origin of the ZnO green luminescence is still not completely understood, it is suggested to be attributed to the zinc vacancies [54]. 

**Figure 10:**
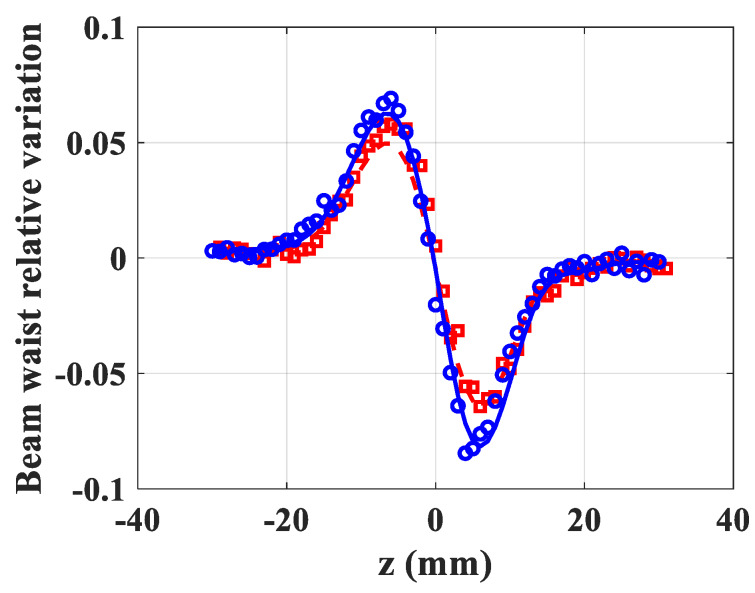
Nonlinear refraction of A and B type suspension of ZnO particles in chloroform at 532 nm (red square: A type; blue circles: B type). Solid and dashed (blue and red) lines are fittings.

The results of the NL optical measurement of ZnO particles suspended in chloroform are listed in Table 1 and Table 2 for a concentration of 1g/l, using excitations at 532 nm and 355 nm, respectively. At 532 nm and for types A and B, the NL absorption is insignificant, and below the resolution of the measuring system (Table 1). The results indicate that *n*_2_ coefficients revealed similar values as that obtained in chloroform solution, while considering the measurement uncertainties (although there is a slight decrease in *n*_2_ for type A). The NL refraction profiles of A and B type related to the particles suspended in chloroform are shown in Figure 10. The data and the corresponding simulation of the D4σ Z-scan fitting of both the BWRV profiles indicate the positive NL refractive responses. It should be mentioned that the main responses are due to the fused silica cell and to the solvent effect, while both materials reveal positive *n*_2_. The peak–valley shape of BWRV (unlike the signature generated with classic Z-scan profiles) informs, that a self-focusing effect (positive NL refractive index) takes place in the studied solutions. As mentioned above, the values of *n*_2_ given in Table 1 are those corresponding to the solutions alone inside the fused silica cell where the response of the glass was considered. The *n*_2_ value $\left(6.2\pm 1.0\right)\times 10^{-20}\;m^{2}/W$ was obtained for pure solvent under the same experimental conditions (wavelength, pulse duration, intensity). 

**Figure 11:**
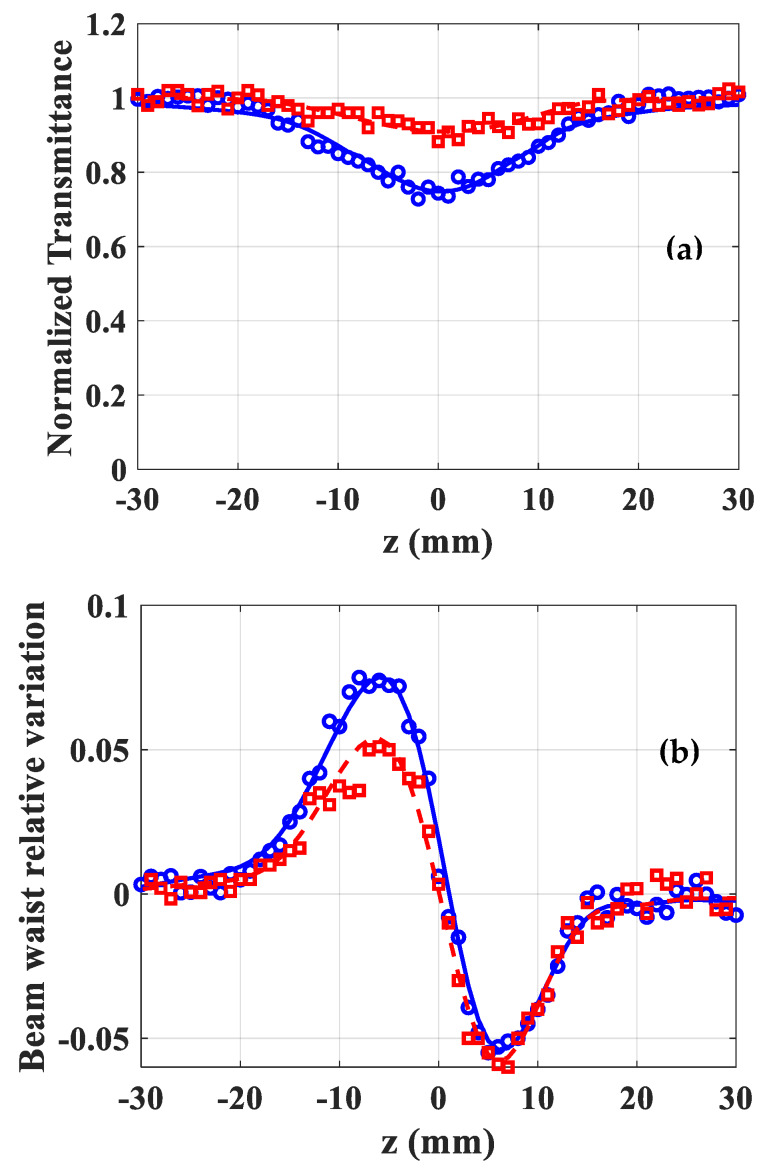
Nonlinear absorption (**a**) and refraction (**b**) of A and B-type suspension of ZnO particles in chloroform at 355 nm (red square: A-type; blue circles: B-type). Solid and dashed (blue and red) lines are fittings.

At 355 nm, the NL absorption coefficient is not negligible even for the solvent (β=0.15 cm/GW). The normalized transmittance profiles, of the nonlinear absorption of both ZnO types (Figure 11a) revealed the significant increase of β for B component, when compared to type A, or to pure solvent. These results clearly confirm the structure differences of A and B particles, at this wavelength. The latter indicated the NL refractive response of the two mixtures (Figure 11b), because *n*_2_ value measured for B is twice *n*_2_ for A. 

In summary, the NL response at 355 nm for both morphologies is higher than measured at 532 nm. Considering the absolute values, one can notice that the NL refractive index of ZnO NPs with modified morphology (cylindrical shaped particles—type B) should be about two times higher than that of ZnO particles with preserved morphology (type A). The latter can be related to the observed enhanced green luminescence of ZnO particles. This result is similar to the one reported for other luminescent nanoparticles (graphene quantum dots) [55], where nonlinear coefficients were significant only at 355 nm, i.e., very close to the linear absorption band. Additionally, no NL response was present in the visible or the near IR range. 

Concluding the synthesis of ZnO particles, we would like to explain that despite the many experiments performed in order to elaborate the shape determining synthesis conditions, we were unable to find parameters which have main impact on formation of the type A and type B particles and it will be the subject of further research.

### 3.2. Thin Layer Fabrication and Characterization

**Figure 12:**
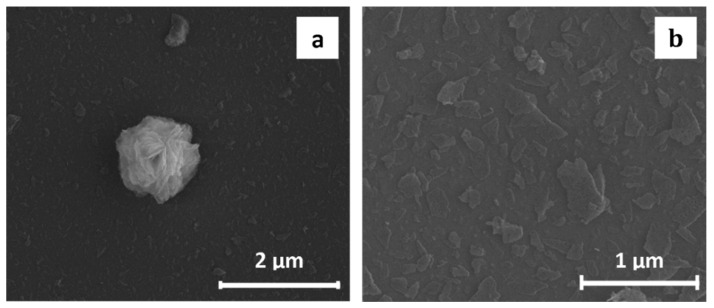
SEM images of ZnO thin film deposited by dip-coating (concentration: 0.3% (w/v); speed: 20 mm/min; reps: 60) and calcinated at 600 °C for 1 h: (**a**) residual ball-like particle, (**b**) uniform layer.

**Figure 13:**
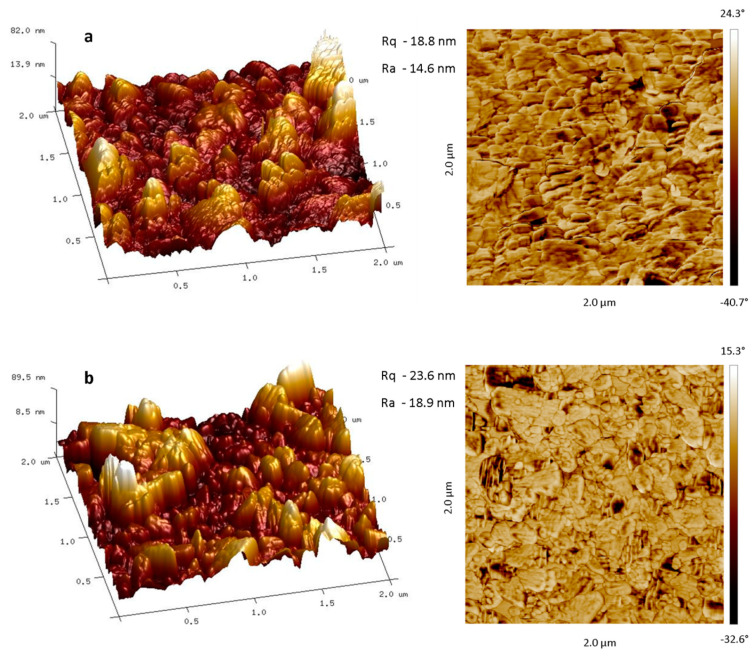
AFM images of 0.3% (w/v) Zn-Carb thin film deposited by dip-coating using the following parameters: (**a**) speed: 20 mm/min, reps: 60; (**b**) speed: 10 mm/min, reps: 60.

ZnO thin films deposited using dip- and spin-coating at various deposition conditions: precursor concentration (0.05–0.3% w/v), deposition speed (5–30 mm/min), number of repetitions (30–60), were calcinated, resulting in the uniform films composed of separated zinc oxide nano-plates at Si substrate (SEM images, Figure 12). AFM images of Zn-Carb films with *R_q_* and *R_a_* parameters (root-mean-square roughness and average roughness) (Figure 13) indicate the lowest surface roughness parameters for films deposited at the highest speed and rep values (Figure 13a).

Prior to PVP deposition on the ZnO layer several trials of polymer spin-coating were carried out in order to control the thickness of the PVP film. For this purpose, PVP ethanol solution (2.5% or 5%) was prepared, stirred for 15 min and used for spin-coating process (conditions listed in Table 3). The thickness of PVP layers were determined by ellipsometric measurements. The layers obtained by means of the spin-coating technique (due to the conditions during deposition) exhibit thickness non-uniformity. This fact was considered during ellipsometric data recording; i.e., apart from the azimuths Ψ and Δ, the depolarization factor (%Depol) was measured. The Ψ and Δ parameters are defined as reported [56,57]

(5) $$\rho \;∼=tan\Psi e^{i\Delta },$$

where ρ∼ is the ratio of the Fresnel reflection coefficients for two orthogonal components of the electric field of the polarized light beam reflected from the surface [56,57]. The depolarization factor, related to the thickness non-uniformity or patterned substrates can be calculated from the following formula [56,57]

(6) $$\%Depol=100\%\left(1-\alpha^{2}-\beta^{2}-\gamma^{2}\right),$$

where: α=cos(2Ψ), β=sin(2Ψ)cos(Δ), and γ=sin(2Ψ)sin(Δ). 

For isotropic non-depolarizing samples, the value of %Depol equals zero. To determine thicknesses of the synthesized PVP films, as well as their optical constants, the four-medium (Si\SiO_2_\PVP\ambient) optical model of a sample was considered. The refractive index *n*(*λ*) of the PVP film was parameterized using a Sellmeier-type dispersion relation [56,57]

(7) $$n^{2}=\varepsilon_{\infty }+\frac{A_{0}}{E_{0}^{2}-E^{2}}$$

In Equation (4), $\varepsilon_{\infty }$=1, *A*_0_, *E*_0_, and *E* are the high-frequency dielectric constant, magnitude, oscillator energy, and photon energy, respectively. The optical constants of Si and SiO_2_ were taken from the database of optical constants [56]. It was assumed that the PVP films summarized in Table 3 exhibit the same optical constants. Therefore in the fitting procedure using the WVASE32^®^ software (J.A. Woollam Co., Inc., Lincoln, NE, USA), the multiple sample analysis approach was applied [56,57,58,59,60]. The model quantities were varied to minimize the standard reduced mean squared error $\chi^{2}$ [56,57].

**Figure 14:**
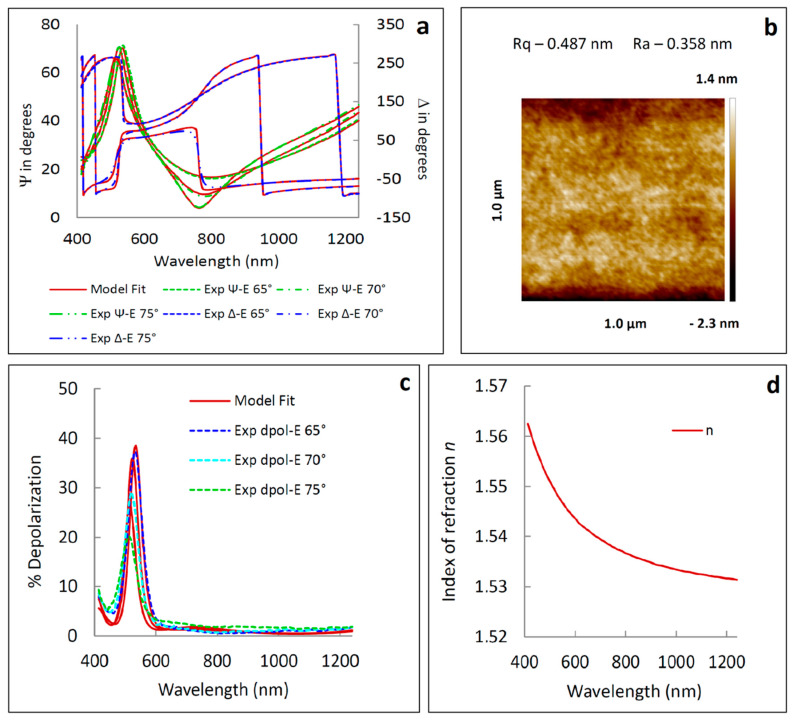
(**a**,**c**) The measured and calculated Ψ and Δ azimuths and depolarization factor %Depol for Si/SiO_2_/PVP No.2 (Table 3). The value of χ^2^ = 10.6. (**b**) The AFM image of Si/SiO_2_/PVP no. 2 (Table 3). (**d**) The refractive index (*n*) of the PVP film.

The measured Ψ and Δ azimuths and depolarization factor %Depol, and sample optical model data are presented in Figure 14a,c. The values of the refractive index, in the measured spectral range, are between 1.532 and 1.563 (for *A*_0_ = 161.4 ± 0.3 eV^2^), whereas the shape of *n* exhibits normal dispersion relation (Figure 14d). Determined thicknesses of PVP films in the range 133–349 nm (Table 3), indicate the tendency to decrease with the concentration decrease and speed rate increase. The proportional relationship between film thickness and spin coating parameters, as well as with solution concentration, has been demonstrated in many studies on polymer thin films [61,62,63]. The non-zero values of %Depol (Figure 14c) confirm the assumption, that the layers demonstrate non-uniform thickness. The coatings prepared at higher concentrations exhibit the lower value of thickness non-uniformity (about 9%), in relation to twice diluted solutions (13–14%). Moreover, the roughness parameters (*R_a_* = 0.49 nm and *R_q_* = 0.36 nm; Figure 14b) indicate quite smooth surface of PVP films. This fact confirms the validity of omission of the rough layer in the optical model of a sample.

**Figure 15:**
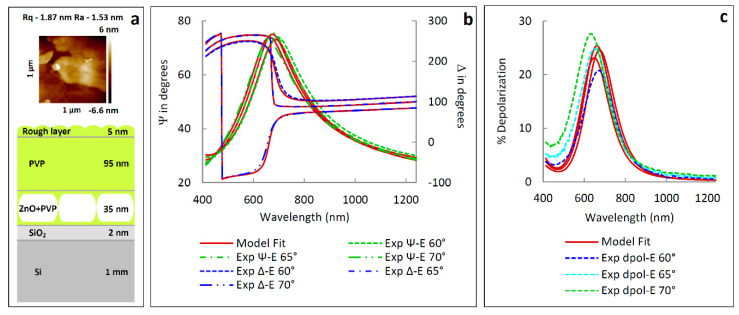
(**a**) AFM image and (**b**) optical model of the ZnO/PVP multilayer system model. (**c**) The measured and calculated Ψ and Δ azimuths as well as depolarization factor %Depol for the Si/SiO_2_/ZnO + PVP sample. The value of χ^2^ = 29.

In the next experiment ZnO layer was covered by the PVP film using conditions in which the thinnest film was obtained (sample **1**). The surface roughness parameters (*R_a_* and *R_q_*) are equal to 1.53 nm and 1.87 nm respectively, what is evident from the AFM images (Figure 15a). These values are 3–4 times larger than the one obtained for the pure PVP films (Figure 14b). Due to the latter for ellipsometric measurements the rough layer was considered. The model of the ZnO/PVP multilayer system (Si\SiO_2_\ZnO + PVP\PVP\rough layer\ambient) is presented in Figure 15b. During the deposition of PVP on ZnO NPs-coated Si the polymer filled the spaces between the ZnO plates. Thus, the coating assembled on the Si substrate can be divided into two layers ZnO NPs + PVP and pure PVP film. To describe optical constants of the ZnO NPs + PVP film ($n_{ZnO+PVP}$) and the rough layer ($n_{rough}$) the Bruggeman type of effective medium approximation (EMA) model was used [56,57]

(8) $$\left(1-f_{PVP}\right)\frac{n_{ZnO}^{2}-n_{ZnO+PVP}^{2}}{n_{ZnO}^{2}+2n_{ZnO+PVP}^{2}}+f_{PVP}\frac{n_{PVP}^{2}-n_{ZnO+PVP}^{2}}{n_{PVP}^{2}+2n_{ZnO+PVP}^{2}}=0,$$

(9) $$f_{ambient}\frac{n_{ambient}^{2}-n_{rough}^{2}}{n_{ambient}^{2}+2n_{rough}^{2}}+\left(1-f_{ambient}\right)\frac{n_{PVP}^{2}-n_{rough}^{2}}{n_{PVP}^{2}+2n_{rough}^{2}}=0,$$

In Equations (5) and (6), *f_PVP_* and *f_ambient_* are the fraction of PVP in the ZnO + PVP film and fraction of void in the rough film, respectively, while *n_ZnO_*, *n_PVP_* and *n_ambient_* are optical constants (the refractive index) of ZnO (taken from [56]), PVP (taken from the previous experiment; Figure 14d) and void, respectively. The value of *f_ambient_* was set to 0.5. The recorded ellipsometric azimuths and depolarization factor for the ZnO/PVP multilayer system and as well as data obtained from the six-medium optical model of a sample are presented in Figure 15b,c. The thicknesses of ZnO + PVP and PVP films determined from ellipsometric data are: 35 ± 4 nm and 95 ± 4 nm, respectively. The thickness of the rough layer 5 ± 3 nm is comparable to the value of the maximum roughness, while the thickness non-uniformity is 19.9 ± 0.2%. The sum of the two above-mentioned thicknesses (130 ± 8 nm) appeared to be close to the value for the separate PVP film—131 nm (the PVP film No. 1 in Table 3). The high thickness of the PVP fraction in the ZnO NPs + PVP layer (85 ± 4%) indicate the formation of the separated zinc oxide nano-plates (SEM images—Figure 12). 

Presented ZnO/PVP multilayer system can be used in various technologies, because the significant improvements to enhance the performances of polymer bulk heterojunction (BHJ) solar cells is still essential. Reported poly(vnylpyrrolidone) incorporation at the interface of a ZnO layer and polythieno[3,4-b]-thiophene-co-benzodithiophene (PTB7):[6,6]-phenyl C71-butyric acid methyl ester (PC70BM) forms photoactive layer in the inverted polymer solar cells. PVP layer revealed 15% enhancement in power conversion efficiency (PCE) [64].

## 4. Conclusions

Regardless to the conditions of ZnO powders synthesis, different types of particles were obtained: ZnO microparticles with well-preserved morphology, cylindrical shaped particles, and intermediate forms, with the cylindrical shaped particles being observed most often. It seems difficult to propose unequivocally what caused the differences, but we supposed it can be caused by CO_2_ migration during the thermal decomposition of carbonate. The measurements indicated that all types of samples were hexagonal wurtzite crystal structure. The optical properties of particles were correlated with their structure by photoluminescence and NL optical measurements. PL spectra enhancement of NL refractive index enabled the correlation of optical properties with the size of structures. Deposition and calcination of the precursor on Si substrates led to formation of uniform films composed of separated nano-plates. ZnO/Si layers with PVP deposited by spin-coating technique revealed a decrease of thickness along with a concentration decrease and speed rate increase. Fabricated ZnO/PVP multilayer systems revealed a thickness of 130 nm determined by spectroscopic ellipsometry measurements.

## Figures and Tables

**Table 1:** Results of the nonlinear optical measurement of ZnO particles in chloroform at 532 nm.

Sample	α (cm^−1^)	E (µJ)	I_0_ (GW/cm^2^)	*n*_2_ × 10^−20^ (m^2^/W)	*β* (cm/GW)
chloroform	0	15	92	6.25 ± 1.0	< 0.004
A-type	0.42	15	95	4.6 ± 1.0	< 0.004
B-type	1.20	15	92	6.5 ± 1.3	< 0.004

**Table 2:** Results of the nonlinear optical measurement of ZnO particles in chloroform at 355 nm.

Sample	α (cm^−1^)	E (µJ)	I_0_ (GW/cm^2^)	*n*_2_ × 10^−20^ (m^2^/W)	*β* (cm/GW)
chloroform	0	5	65	7.5 ± 2.6	0.15 ± 0.05
A-type	0.59	5	64	5.5 ± 2.5	0.08 ± 0.02
B-type	2.12	5	66	9.6 ± 2.7	0.31 ± 0.10

**Table 3:** Spin-coating conditions of PVP films, film thickness, and thickness non-uniformity.

No.	PVP Concentration (%)	Spin-Coating Parameters	Film Thickness (nm)	Thickness Non-Uniformity (%)
1	2.5	Step 1: 5000 rpm 30 s Step 2: 5000 rpm 30 s	133 ± 1	13.1 ± 0.4
2	5		317 ± 1	8.8 ± 0.1
3	2.5	Step 1: 2000 rpm 20 s Step 2: 5000 rpm 30 s	138 ± 1	14.2 ± 0.4
4	5		349 ± 1	8.7 ± 0.2
